# A Reappraisal of Sedimentation Nonideality Coefficients for the Analysis of Weak Interactions of Therapeutic Proteins

**DOI:** 10.1208/s12248-019-0307-0

**Published:** 2019-02-27

**Authors:** Sumit K. Chaturvedi, Peter Schuck

**Affiliations:** Dynamics of Macromolecular Assembly Section, Laboratory of Cellular Imaging and Macromolecular Biophysics, National Institute of Biomedical Imaging and Bioengineering, National Institutes of Health, 13 South Drive, Bldg. 13, Rm 3N17, Bethesda, Maryland 20892 USA

**Keywords:** hydrodynamics, protein-protein interactions, protein-solvent interactions, sedimentation velocity, virial coefficients

## Abstract

The study of weak or colloidal interactions of therapeutic proteins in different formulations allows prediction and optimization of protein stability. Various biophysical techniques have been applied to determine the second osmotic virial coefficient *B*_*2*_ as it reflects on the macromolecular distance distribution that governs solution behavior at high concentration. In the present work, we exploit a direct link predicted by hydrodynamic theory between *B*_*2*_ and the nonideality of sedimentation, commonly measured in sedimentation velocity analytical ultracentrifugation through the nonideality coefficient of sedimentation, *k*_*S*_. Using sedimentation equilibrium analytical ultracentrifugation for independent measurement of *B*_*2*_, we have examined the dependence of *k*_*S*_ on *B*_*2*_ for model proteins in different buffers. The data exhibit the expected linear relationship and highlight the impact of protein shape on the magnitude of the nonideality coefficient *k*_*S*_. Recently, measurements of *k*_*S*_ have been considerably simplified allowing higher throughput and simultaneous polydispersity assessment at higher protein concentrations. Thus, sedimentation velocity may offer a useful approach to compare the impact of formulation conditions on weak interactions and simultaneously on higher-order structure of therapeutic proteins.

## INTRODUCTION

Protein pharmaceuticals are a rapidly expanding class of therapeutics. In particular, monoclonal antibody products have found broad application with an increasing number of targets (1–3). One of the challenges is the development of formulations that provide colloidal stability, low solution viscosity, and ensure the absence of immunogenic aggregates at very high protein concentrations (4–7). Therefore, techniques that can assess both the formation of higher-order structure and measure weak particle interactions are of great interest in this field. In particular, it has been shown that the second virial coefficient *B*_*2*_ can correlate with aggregation, viscosity, solubility, and liquid-liquid phase separation (8–15). Techniques used to measure *B*_*2*_ include small angle scattering, static light scattering (SLS), sedimentation equilibrium (SE) and sedimentation velocity (SV) analytical ultracentrifugation (AUC), self-interaction chromatography, osmometry, and dynamic light scattering (DLS) in combination with SV (9,13,15–26).

Unfortunately, measurement of the second osmotic virial coefficient is challenging with any technique, and difficulties are exacerbated by sample polydispersity. For example, SLS is a very attractive method due to the ability to measure solutions at very high concentrations, even beyond the range where *B*_*2*_ alone is sufficient to describe the concentration-dependent behavior and more precise analyses are necessary (27,28). On the other hand, SLS signals are highly sensitive to trace aggregates and impurities of larger size, which may be encountered in concentrated solutions (10). Another light scattering approach is DLS, where the change in mutual diffusion coefficient *D* with concentration *c* can be measured through the nonideality coefficient of diffusion, *k*_*D*_. As has been pointed out by Saluja and colleagues, through analysis of the autocorrelation function in DLS, it is possible to discriminate and exclude signals from large particles that would dominate SLS measurements (10). However, a caveat is that this does not work for oligomers and smaller aggregates that cannot be resolved in DLS, and that strictly the basic foundations of polydispersity analysis in DLS break down in the presence of hydrodynamic interactions (29). Conflicting results were reported from experiments probing the theoretically expected linear relationship between the nonideality coefficient of diffusion, *k*_*D*_ and *B*_*2*_. While Ghosh *et al.* observed poor correlation between *k*_*D*_ by DLS and *B*_*2*_ by SLS (27), two other studies report good correlations (9,30). However, the relationship was not considered universal but molecular shape dependent (9).

The nonideality coefficient of diffusion and the second virial coefficient are intimately linked to the nonideality coefficient of sedimentation, *k*_*S*_, as measured from the decreasing sedimentation velocity with higher concentrations, through the simple relationship *k*_*D*_ = 2*B*_*2*_–*k*_*S*_ (9,16,27,31,32) (considering *B*_*2*_ in volume/weight units; analogous to *B*_*2*_*′M* with a second virial coefficient *B*_*2*_*′* in molar units). Thus, the combination of DLS to measure *k*_*D*_ and SV to measure *k*_*S*_ has been used to determine *B*_*2*_ (9,10,30).

Previous work in this field has not yet made use of the result from statistical fluid dynamics that hydrodynamic interactions that govern *k*_*S*_ are directly dependent on the interparticle distance distribution, in a way that establishes a direct link between the reduction of sedimentation in nonideal solutions and the second virial coefficient without reference to diffusion nonideality (33–37). The question whether this can be exploited for weakly interacting proteins and to what extent shape-dependent (i.e., protein-dependent) parameters need to be considered in the interpretation of *k*_*S*_ is examined in the present work. Examining weak interactions through nonideal SV seems attractive because, independently, SV is already widely used to quantitate the presence of immunogenic oligomers and higher-order structures in formulations of therapeutic proteins, orthogonal to chromatographic and other methods (29,38–41).

This problem gains more practical relevance since we have recently introduced a new method to determine nonideality coefficients of sedimentation simultaneous with high-resolution size distributions from single experimental data sets (29), which eliminates the need to run samples at multiple concentrations for determining *k*_*S*_. The new method also increases the concentration limits for the quantitation of trace oligomers and aggregates by SV, providing information on higher-order structures closer to formulation conditions from the same experiment. Furthermore, we have recently shown that weak macromolecular self-association can be measured with this approach (42). In parallel, alternative detection modes (43) and the use of 3D printing technology (44) in the development of new centerpieces can enhance the throughput and reduce sample requirements of SV (manuscript submitted). Therefore, we believe the measurement of nonideality coefficients *k*_*S*_ by SV can be an attractive approach for the efficient characterization of weak interactions and higher-order structure of therapeutic proteins in different formulation conditions.

## MATERIALS AND METHODS

### Proteins

NISTmAb (45) was purchased from NIST (SRM 8671) and studied in the original buffer of 25 mM histidine, pH 6.0, or was dialyzed into 25 mM histidine buffer pH 6.0 with 5 mM or 50 mM NaCl, respectively. VRC01 and VRC07-523LS were kindly provided by Drs. Jai Pathak, Lisa Kueltzo, and Frank Arnold. VRC01 was studied in 25 mM Na-Citrate, 50 mM NaCl, 150 mM Arginine HCl, pH 5.8, and VRC07-523LS was studied in 50 mM Histidine, 50 mM NaCl, 5% *w*/*v* sucrose, 2.5% w/*v* sorbitol, pH 6.8. Hen egg lysozyme was purchased from Sigma (catalog # L6876; Sigma Aldrich, St. Louis, MO) and dialyzed into 10 mM acetate buffer pH 4.6 with 10 mM, 100 mM, or 300 mM NaCl, respectively. Ovalbumin was purchased from Sigma (catalog # A5503; Sigma Aldrich, St. Louis, MO) and dialyzed into 10 mM sodium phosphate, pH 7.4, 150 mM NaCl. Protein concentrations were measured refractometrically by Rayleigh interferometry in the analytical ultracentrifuge.

### Analytical Ultracentrifugation

SE and SV experiments were carried out in an Optima XL-A/I (Beckman Coulter, Indianapolis IN) following standard techniques (46,47) unless mentioned otherwise. For SE, cell assemblies were mechanically stabilized by repeated exposure to strong centrifugal fields. Buffer blanks for SE were measured at one or multiple equilibrium rotor speeds (9000 rpm and 14,000 rpm for antibodies; 25,000 rpm or 27,000 rpm or 50,000 rpm for lysozyme; 18,000 rpm for ovalbumin). Samples were filled into cell assemblies with 3 mm or 12 mm pathlength Epon double sector centerpieces at volumes to achieve ≈ 4 mm or ≈ 12 mm solution column heights for SE or SV, respectively. The rotor was temperature equilibrated to a nominal temperature of 19.7°C while resting in the rotor chamber prior to the start of centrifugation. For SE, an overspeeding schedule was calculated to minimize the equilibration time (48); for SV the rotor was accelerated to full speed at once (40,000 rpm for VRC antibodies, 45,000 rpm for NISTmAb, 50,000 rpm for all other). Rayleigh interference optical data acquisition was used. All data analyses were carried out with models implemented in SEDFIT, SEDPHAT (available at sedfitsedphat.nibib.nih.gov), and MATLAB (Mathworks, Natick, MA). Statistical error analyses were based on F-statistics with a *P* value of 0.95 (49) (SE analysis) or via standard χ^2^ error analysis of linear regression (50) (SV analysis).

### Sedimentation Velocity Analysis

With experiments predating the recently introduced nonideal sedimentation coefficient distribution approach (29), all data were analyzed using the traditional two-step procedure: first, scan sets were fitted with the standard sedimentation coefficient distribution *c*(*s*) (51,52). Integration of the monomer peak was carried out to determine the weight-average sedimentation coefficient *s*_*w*_, and, from the data at the lowest protein concentration approximating ideal dilution, the best-fit frictional ratio *f*/*f*_*0*_. This determination of *s*_*w*_ is equivalent to considerations of mass transport through a plane in the plateau region and independent of radial position, normalized to transport conditions at the start of centrifugation (52,53). However, since samples undergo slight radial dilution in the sector-shaped sample (54), the corresponding sample concentration was calculated as the weighted time-average plateau concentration previously introduced for self-associating systems (32,53). An uncertainty arises from the mist-fit of the standard sedimentation coefficient distribution *c*(*s*), and the impact on *s*_*w*_ can be estimated by calculating mass transport implied by residuals of the fit. Because the residuals are largely symmetrical around the inflection point, this misfit typically amounted to errors in *s*_*w*_ values < 10^−3^ S.

In a second analysis step, nonideality coefficients *k*_*S*_ were calculated from *s*_*w*_ as a function of concentration by fitting

1 $$ s(w)=s(0)\left(1-{k}_Sw\right) $$

where *w* denotes the protein weight concentration. (Alternatively, the lysozyme sedimentation scans were fitted with nonideal sedimentation coefficient distribution *c*_NI_(*s*_0_) to determine *k*_*S*_ directly (29,42).) For the purpose of evaluating *k*_*S*_ from the relative reduction of the sedimentation velocity, it is not necessary to normalize sedimentation coefficient data to conditions in water at 20°C (neither is it necessary to apply small calibration factors (55,56) for this purpose). In this regard, it should be noted that an alternate approach of applying solution density corrections leads to an analogous framework but with nonideality coefficients ${k}_S^{\prime }$ reduced by the partial-specific volume $\overline{v}$, as discussed by Harding and Johnson (31). The framework of uncorrected *s* values used in the present work corresponds naturally to the nonideal sedimentation coefficient distributions *c*_*NI*_(*s*_0_) (29). It also matches the statistical fluid mechanics treatment of sedimentation by Batchelor (37,57), which for non-interacting hard spheres in the dilute regime predicts the linear relationship

2 $$ s\left(\varPhi \right)=s(0)\left(1-6.55\varPhi \right) $$

where *Φ* denotes the volume fraction occupied by the sedimenting particle (57). This expression has been generalized by Batchelor and Wen (37) for weakly interacting spheres in the dilute regime

3 $$ s\left(\varPhi \right)=s(0)\left(1-3.03\varPhi -3.52{B}^{\ast}\varPhi \right) $$

with the reduced second virial coefficient B* defined as the ratio of *B*_*2*_ and the hard-sphere virial coefficient *B*_*HS*_ (33–37). Extensions for higher-order approximations suitable for higher volume fractions using a Baxter sticky-sphere approach were presented by Swan and colleagues (36).

It is worth highlighting the fundamental difference between Eq. 3 and the previously widely used relationship between *k*_*S*_ and *B*_*2*_ via the nonideality coefficient of diffusion *k*_*D*_ = 2*B*_*2*_–*k*_*S*_ (9,16,27,31,32). The latter can be derived on the basis of the virial expansion of the osmotic susceptibility, and therefore rests on thermodynamic considerations where no detailed molecular model is invoked, and where coefficients *k*_*S*_ and *k*_*D*_ may be considered phenomenological parameters of concentration dependence. By contrast, Eq. 3 rests on statistical fluid mechanics and the explicit calculation of hydrodynamic forces between sedimenting spheres, based on the particle distance distribution. This distance distribution is related to the interparticle potential and therefore linked to the second virial coefficient. It accounts, for example, for the probability of transient dimer formation (close proximity of two particles) that would diminish overall hydrodynamic drag (37,58). Therefore, Eq. 3 does not require a reference to *k*_*D*_.

To relate the linear coefficients in Eqs. 1 and 3, it is necessary to consider the connection between protein concentration and the relevant volume fraction. This is far from trivial and a long-standing problem for non-globular macromolecules (31,59). If only the macromolecular volume excluded from solution is considered, the volume fraction relates directly to the protein partial-specific volume, $\overline{v}$ and with $\varPhi =w\overline{v}$ one arrives at a prediction for the nonideality coefficient of sedimentation

4 $$ {k}_{S,V}\sim \left(3.03+3.52\left[{B}_2/{B}_{HS}\right]\right)\overline{v} $$

However, theoretical work for nonideality coefficients of non-spherical particles have revealed a particle shape dependence (31,60–62), and a large body of experimental work in SV has shown that nonideality coefficients of proteins strongly increase with macromolecular shape asymmetry (62,63). In his theory of nonideal sedimentation, Rowe has proposed the relevant volume fraction to be that of the hydrodynamically equivalent sphere (32,64), which leads to the relationship

5 $$ {\varPhi}_S=w\overline{v}{\left(f/{f}_0\right)}^3 $$

with *f/f*_*0*_ denoting the protein frictional ratio, such that *Φ*_*S*_ represents the total volume occupied by hydrodynamically equivalent spheres. We insert this consideration into the framework of Batchelor’s relationships. In this approximation, the nonideality coefficient for sedimentation would be expected to follow

6 $$ {k}_{S,H}\sim \left(3.03+3.52\left[{B}_2/{B}_{HS}\right]\right)\overline{v}{\left(f/{f}_0\right)}^3 $$

as a function of both frictional ratio and second virial coefficient.

Whether relationship Eq. 4 or Eq. 6 provides a better description of experimental data for proteins is not clear. The attempt to use experimental *k*_*S*_ values to decide between hydrodynamic volume models is complicated by the usually unknown contribution of particle interactions to *B*_*2*_. Compounding these problems, it should be noted that for non-spherical particles *B*_*HS*_ will also be a non-trivially shape-dependent quantity (65). Still, we may unify both expressions Eqs. 4 and 6 into the form

7 $$ {k}_S={k}_S^0\left(1+1.16\left[{B}_2/{B}_{HS}\right]\right) $$

Under “theta conditions” where *B*_*2*_ vanishes, the nonideality coefficient of sedimentation termed ${k}_S^0$, will be a reflection of the relevant hydrodynamic volume alone. In this way, the data sets of *k*_*S*_ vs. *B*_*2*_ should offer the opportunity to shed more light on this question. But in any event, the nonideality coefficient increases linearly with the second virial coefficient, which may be sufficient for comparative screening applications even without determining the absolute values of *B*.

### Sedimentation Equilibrium Analysis

The radial concentration distributions of a single species in sedimentation equilibrium follow

8 $$ c(r)=c\left({r}_0\right)\exp \left[\frac{M}{1+c(r)\left(\partial \ln \gamma /\partial c\right)(r)}\times \frac{\left(1-\overline{v}\rho \right){\omega}^2}{RT}\left({r}^2-{r}_0^2\right)\right] $$

where *M* is the molar mass, *γ* the chemical activity coefficient, *R* the gas constant, *T* the temperature, *ρ* the solvent density, *ω* the rotor angular velocity, and *r*_*0*_ a reference radius (21,66). At not too high concentrations, accounting only for the first term of the virial expansion of the activity coefficient leads to the implicit equation for the measured signal *a*(*r*)

9 $$ a(r)=c\left({r}_0\right)\exp \left[\frac{M}{1+2{B}_2^{\prime } Mc(r)}\times \frac{\left(1-\overline{v}\rho \right){\omega}^2}{RT}\left({r}^2-{r}_0^2\right)\right]+b(r) $$

which can be solved iteratively and may be superimposed by a baseline term *b*(*r*). Alternatively, to account for a wider concentration range, the chemical activity as a function of occupied volume fraction may be approximated with the Carnahan-Starling formula

10 $$ \ln \gamma =\frac{8\varPhi -9{\varPhi}^2+4{\varPhi}^3}{{\left(1-\varPhi \right)}^3} $$

for suspensions of hard spheres (67,68). Considering the protein as effective hard spheres of swollen radius with $\varPhi = cM\overline{v}\left({B}_2/{B}_{HS}\right)$ and with the virial coefficient of non-interacting hard spheres ${B}_{HS}=4\overline{v}$_,_ we can extend Eq. 9 by inserting

11 $$ \frac{\partial \ln \gamma }{\partial c}=M\overline{v}\frac{B_2}{B_{HS}}\left[\frac{24\varPhi -27{\varPhi}^2+12{\varPhi}^3}{{\left(1-\varPhi \right)}^4}+\frac{8-18\varPhi +12{\varPhi}^2}{{\left(1-\varPhi \right)}^3}\right] $$

in Eq. 8 (68). At low concentrations, it has the limiting value $8M\overline{v}\left({B}_2/{B}_{HS}\right)$, which is consistent with the value of $2M{B}_2^{\prime }$ as in Eq. 9. At a volume fraction of 1%, the difference to Eq. 9 amounts to ≈ 4% in *B*_*2*_, increasing approximately proportionally to a volume fraction of 5%. Equation 11 was implemented in SEDPHAT using the INVEQ approach (21). This model was fitted globally to multi-speed data sets at multiple loading concentrations, weighted with inverse loading concentration (69).

## RESULTS

**Fig. 1:**
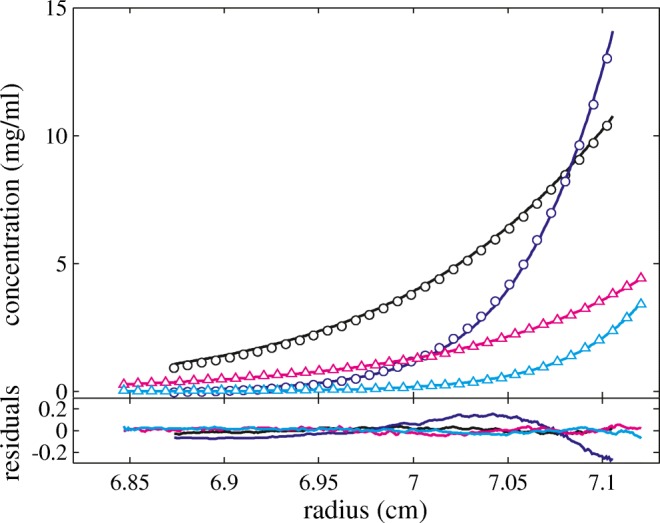
SE data of NISTmAb in 25 mM histidine pH 6.0 with 5 mM NaCl. Shown are Rayleigh interferometric fringe shift data in a 3 mm pathlength cell at 9000 rpm and 14,000 rpm (black and blue circles, respectively) and at much lower loading concentration in a 12 mm pathlength cell at the same rotor speeds (magenta and cyan triangles, values tenfold magnified). Only every 10th data point is shown for clarity. The solid lines are the respective best-fit distribution based on a global analysis of data from 4 different cells. The best-fit buoyant molar mass is 39.2 kDa (corresponding to a ≈ 150 kDa protein) and the best-fit virial coefficient is 11.6 (10.7–12.4) ml/g. The lower panel shows the residuals from the fit with an rmsd of 0.029 fringes

**Fig. 2:**
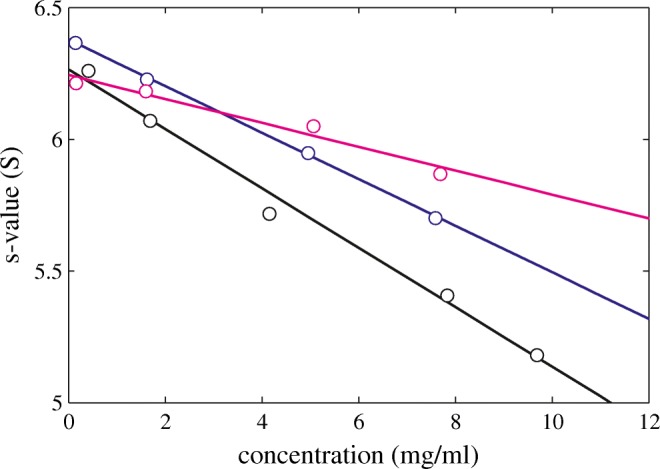
Experimental sedimentation coefficients of NISTmAb monomer at different conditions. All data are in 25 mM histidine pH 6.0 with 0 (black), 5 mM (blue), or 50 mM NaCl (magenta). The effective concentrations were calculated from the loading concentrations corrected for slight dilution during the SV run. The line represents the best-fit linear regression. Deviations from the expected straight line fit were used to estimate errors in *s*_*w*_ which were propagated to uncertainties in *k*_*S*_ (50)

We chose the NISTmAb reference molecule in different buffers to examine the nonideality coefficient of sedimentation *k*_*S*_ and its relationship to the second virial coefficient *B*_*2*_. For NISTmAb in 25 mM histidine pH 6.0 (in the formulation buffer that does not contain salt) from SE experiments, we obtained a best-fit estimate for *B*_*2*_ of 27.1 ml/g (25.5–28.7 ml/g; 95% confidence interval), which compares well with the value of 25 ml/g reported by others using SLS and SE in these conditions (70). Figure 1 shows typical multi-speed SE data for NISTmAb up to 13 mg/ml in 5 mM NaCl, with the best-fit single nonideal species sedimentation model Eq. 8 based on effective hard spheres Eq. 11. SV data for the same set of conditions are shown in Fig. 2, with the best-fit linear regression to determine *k*_*S*_ following Eq. 1. The estimated variance of the *s*_*w*_ values was 0.03 S, slightly above the expected experimental precision of 0.01 S.

**Fig. 3:**
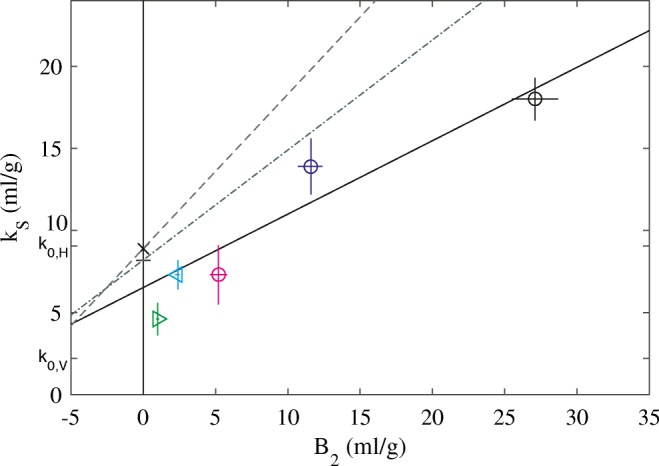
Relationship between measured *k*_*S*_ and *B*_*2*_ for the NISTmAb and VRC antibodies studied. Shown are the experimental data points for NISTmAb in formulation condition (black circle) and supplemented with 5 mM (blue circle) or 50 mM NaCl (magenta circle). The solid line is the linear regression of NISTmAb data (R = 0.93). Additionally shown are data points for VRC01 (cyan triangle) and VRC07-523LS (green triangle). The vertical line indicates the condition where the virial coefficient vanishes and does not make any contribution to the hydrodynamic nonideality of sedimentation (theta condition). The intercept of this line with the interpolated regression may be compared with the theoretical predictions *k*_*0,V*_ (Eq. 4) and *k*_*0,H*_ (Eq. 6) of accounting for the occupied volume fraction on the basis of the protein partial-specific volume only, or considering the hydrodynamically equivalent spheres, respectively. The *k*_*S*_ values calculated based on the regression of the *k*_*D*_ vs. *B*_*2*_ data of different antibodies measured by DLS/SLS in Lehermayr *et al.* (30) is shown as short dashed line, and the ones based on a similar regression of different antibodies measured by DLS/SV in Connolly *et al.* (9) are shown as short dotted line, with the respective values at the theta condition highlighted as cross and plus sign, respectively

The nonideality coefficients are plotted against *B*_*2*_ in Fig. 3 (circles). Within error, they follow the expected linear relationship. Two additional antibodies were studied, VRC01 and VRC07-523LS, shown as cyan and green triangles in Fig. 3. Within error, they also fall on the same regression line from NISTmAb, suggesting shape-dependent factors to be consistent between these antibodies.

It is possible to examine the hydrodynamic volume in more detail. From the NISTmAb SV data at the lowest protein concentration, which are sufficiently dilute for the ideal sedimentation model to apply, frictional ratios of ≈ 1.6 were obtained for all conditions. Together with a protein partial-specific volume of 0.73 ml/g, this allows us to calculate the expected *k*_*S*_ value for conditions of vanishing virial coefficients (theta condition) considering only the protein partial-specific volume (Eq. 4), termed *k*_*0,V*_, or the analogous value considering the volume of the hydrodynamic equivalent sphere, termed *k*_*0,H*_. The values for *k*_*0,V*_ and *k*_*0,H*_ are labeled on the ordinate axis of Fig. 3. From interpolation of the experimental NISTmAb data points to the theta conditions *B*_*2*_ = 0 we can derive an experimental estimate of the nonideality of sedimentation for vanishing virial coefficients, ${k}_S^0$ (Eq. 7), which exclusively reports on the shape dependence of the hydrodynamic interactions. As may be discerned from Fig. 3, the experimental value is 6.5 ml/g, i.e., in between *k*_*0,V*_ (2.2 ml/g) and *k*_*0,H*_ (9.1 ml/g). The experimental value represents a ≈ 3-fold volume expansion relative to the merely displaced volume, but only 72% the volume of the hydrodynamically equivalent spheres.

Interestingly, at this theta point *k*_*D*_ = −*k*_*S*_, i.e., the nonideality coefficient of diffusion and sedimentation must be of the same magnitude and opposite sign. Therefore, the linear regression of the relationship between *k*_*D*_ and second virial coefficient among different antibodies reported by Connolly (9) and those by Lehermayr (30) may be compared at the theta condition, leading to ${k}_S^0$ values of 8.2 ml/g and 8.9 ml/g, respectively (indicated as cross and plus sign in Fig. 3). Similar ${k}_S^0$ values at theta conditions of 8–10 ml/g can be estimated from the study of different antibodies at different solution conditions by Saito and colleagues (15). These values are all close to the value of 9.1 ml/g predicted for the hydrodynamically equivalent sphere of antibodies. It is possible to further transform the regressions *k*_*D*_ vs. *B*_*2*_ reported by Connolly (9) into *k*_*S*_ vs. *B*_*2*_ noting that *k*_*S*_ = 2*B*_*2*_–*k*_*D*_. As can be discerned from the dotted and short dotted lines in Fig. 3, such transforms are consistent with the present data neither for those measured by Connolly *et al.* using DLS/SV (9) nor with those measured by Lehermayr based on DLS/SLS (30).

Next, we tested these relationships with a smaller and more compact protein, using hen egg lysozyme as another well-studied model protein. The virial coefficients *B*_*2*_ were measured again by SE in different solution conditions, all at pH 4.6 in 10 mM acetate, with different degree of charge screening by 10 mM NaCl, 100 mM NaCl, and 300 mM NaCl. Many groups have previously reported decreasing *B*_*2*_ with increasing salt (71–73) and our experimental *B*_*2*_ values compare favorably with the range of published values (10,13,72).

**Fig. 4:**
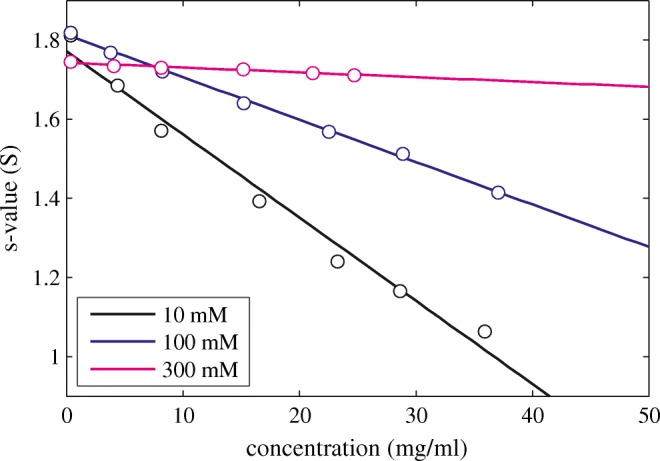
Experimental sedimentation coefficients of lysozyme at different conditions. All data are at pH 4.6 in 10 mM acetate with different concentration of NaCl: 10 mM NaCl (black), 100 mM NaCl (blue), and 300 mM NaCl (magenta). The line represents the best-fit linear regression based on Eq. 1

**Fig. 5:**
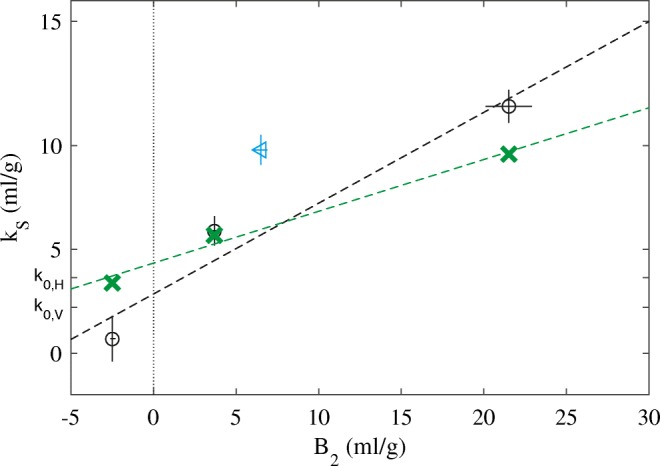
Relationship between measured k_S_ and B_2_ for hen egg lysozyme. Shown are the experimental data points for lysozyme in 10 mM sodium acetate pH 4.6 with 10 mM, 100 mM, or 300 mM NaCl (circles). The dashed black line is the linear regression (*R* = 0.97). Indicated on the ordinate are the theoretical predictions *k*_*0,V*_ and *k*_*0,H*_ at the theta condition when accounting for the occupied volume fraction on the basis of the protein partial-specific volume only, or considering the hydrodynamically equivalent spheres, respectively. The green data points reflect *k*_*S*_ values derived from a sedimentation coefficient distribution *c*_NI_(*s*_0_) that accounts separately for polydispersity from lysozyme self-association (taken from (42) for the 100 mM and 300 mM NaCl conditions), with linear regression (*R* = 0.992; dashed green line). Additionally shown are data from ovalbumin in PBS (cyan triangle)

The SV measurements to determine *k*_*S*_ are shown in Fig. 4. The plot *k*_*S*_ vs. *B*_*2*_ (Fig. 5) shows the expected strong correlation. Due to the more compact shape (best-fit *f/f*_*0*_ = 1.18) the *k*_*0,V*_ and *k*_*0,H*_ are closer together (2.2 ml/g and 3.6 ml/g, respectively), with the interpolated experimental value of ${k}_S^0$ 2.9 ml/g in between the two values.

Lysozyme is known to self-associate dependent on solution pH and ionic strength, a feature also exhibited by some therapeutic antibodies and other therapeutic proteins (74,75). Self-association is a reflection of attractive protein-protein interaction and as such well described in the framework of virial coefficients (76–78), which become smaller and even negative, as is the case for lysozyme at pH 4.6 in 300 mM NaCl. Similarly, *k*_S_ values decrease as a result of self-association (79). In the case of moderate and strong self-association, due to poor convergence of virial expansion (76), the concentration-dependence of equilibrium properties is better described by explicitly accounting for different oligomeric states in chemical equilibrium. Analogously, it is possible to switch the hydrodynamic analysis to a picture that allows to describe polydispersity from self-association separately (42). In this alternative picture, *k*_S_ is determined, not from the concentration-dependence of the weight-average *s* value, but from the nonideal sedimentation coefficient distribution *c*_NI_(*s*_0_). These resulting *k*_*S*_ data (shown in green in Fig. 5, taken from (42)) are found also to exhibit a linear relationship with *B*_*2*_.

Finally, a single experiment with ovalbumin in PBS is shown in Fig. 5 as cyan triangle. This protein has a different structure and a different shape factor (with a best-fit frictional ratio of 1.24) and it does not fall on the same line as either lysozyme or NISTmAb. Similarly, neither the experimental *k*_*S*_-*B*_*2*_ relationships of lysozyme nor NISTmAb are consistent with each other. This supports the notion that the precise relationship will be dependent on the specific protein shape.

## DISCUSSION

The choice of techniques for measuring the second virial coefficient depends on many factors, including purity, experimental time, concentration range, and volumes. In the present work, we have focused on AUC approaches. Among all techniques for measuring the second virial coefficient, traditional analytical ultracentrifugation may be one of the lowest throughput methods. However, a significant advantage is the intrinsic size separation: large particles will quickly sediment and not impact the measurement. This makes it a very attractive approach for samples that might potentially contain traces of higher-order structure oligomers or particles.

As Saito and co-workers (15) have pointed out, SE is attractive due to its relatively small sample volumes and the ability to study higher concentrations than the loading concentration due to the accumulation of protein in the distal end of the solution column afforded by the gravitational field. In order to facilitate SE experiments, we have previously introduced a time-varying centrifugal field approach that can shorten the time required to reach equilibrium (48). In the present, work we have implemented a new sedimentation model that allows determination of *B*_*2*_ across a wider concentration range. It accounts for higher-order virial coefficients through relationships based on effective hard spheres, and due to the higher concentration, the range should provide higher precision in *B*_*2*_.

The main focus of the present communication is to highlight an opportunity to measure virial coefficients directly by SV exploiting a relationship from statistical fluid mechanics that predicts the magnitude of *k*_*S*_ in the presence of weak particle interactions. Within error, our data confirm the linear relationship between *k*_*S*_ and *B*_*2*_. They also highlight the difficulty of quantitatively interpreting the absolute magnitude of *k*_*S*_, due to uncertainties in the relevant hydrodynamic volume, although our data suggest the volume of the Stokes (hydrodynamic equivalent) sphere may be the best estimate. More precise studies in this area would be plagued by the impossibility to vary solution conditions without simultaneously varying the effective sedimenting particle including hydration, sedimenting counter-ions, and conformation (80), and may be limited by simplified models of particle shapes (80,81). The interpretation of the absolute value of *k*_*S*_, even for spherical particles such as polystyrene latex beads (59), is non-trivial due to the unknown contributions of weak interactions (82).

Pragmatically, however, questions regarding the absolute values of *k*_*S*_ should not impact the application of SV to screen and compare different buffer conditions. One could argue that absolute values for *B*_*2*_ may be hard to dissect into steric contributions and those from weak interactions and that relative values *B*_*2*_/*B*_*HS*_ may sufficiently reflect on the differences of weak interactions in different buffer conditions. Finally, it appears that if *k*_*D*_ may be used to assess differences in *B*_*2*_ and exhibits a linear relationship, as proposed by Connolly and colleagues (9), then *k*_*S*_ must likewise exhibit a linear relationship with *B*_*2*_ (since *k*_*S*_ = 2*B*_*2*_–*k*_*D*_). Our data support the conclusion by Connolly and colleagues that *k*_*S*_ values should be compared only for the same molecule. Which technique provides better approach may depend on several factors including relative accuracy and sample properties. Two very recent developments have significantly enhanced SV. One is the ability to measure in a single experiment at high concentration (e.g., up to 80 mg/ml of γ-crystallin) of unlabeled material the nonideality coefficient alongside the sedimentation coefficient distribution (29). This is synergistic with the application of SV to quantify higher-order structures, such as oligomers and aggregates, in different conditions (29,38–41). Second, we have developed three-channel centerpieces that allow doubling the capacity of SV samples in an AUC run and using smaller sample volumes (manuscript submitted). This can greatly improve the throughput of SV, extending the number of conditions that can be examined in a single run from 1 or 2 to up to 15. 

As pointed out by Roberts and co-workers (28), there are inherent limitations of measuring protein properties in a relatively low concentration regime to predict behavior at much higher concentration. In future work, making use of recent developments in theory describing sedimentation at higher concentrations beyond the first-order nonideality approximation (36), detection technology (83–85), and centerpiece design (44) (manuscript in preparation), it may be possible to extend SV experiments further to highly concentrated formulation conditions measuring both weak interactions and sample polydispersity.
